# Multiscale divergence between Landsat- and lidar-based biomass mapping is related to regional variation in canopy cover and composition

**DOI:** 10.1186/s13021-018-0104-6

**Published:** 2018-09-14

**Authors:** David M. Bell, Matthew J. Gregory, Van Kane, Jonathan Kane, Robert E. Kennedy, Heather M. Roberts, Zhiqiang Yang

**Affiliations:** 1 Pacific Northwest Research Station, USDA Forest Service, 3200 SW Jefferson Way, Corvallis, OR 97331 USA; 2 Forest Ecosystems and Society Department, Oregon State University, Corvallis, OR USA; 3 School of Environmental and Forest Sciences, University of Washington, Seattle, WA USA; 4 College of Earth, Ocean, and Atmospheric Sciences, Oregon State University, Corvallis, OR USA

**Keywords:** Aboveground live biomass, Forest, Landsat, Lidar, Prediction, Aggregation

## Abstract

**Background:**

Satellite-based aboveground forest biomass maps commonly form the basis of forest biomass and carbon stock mapping and monitoring, but biomass maps likely vary in performance by region and as a function of spatial scale of aggregation. Assessing such variability is not possible with spatially-sparse vegetation plot networks. In the current study, our objective was to determine whether high-resolution lidar-based and moderate-resolution Landsat-base aboveground live forest biomass maps converged on similar predictions at stand- to landscape-levels (10 s to 100 s ha) and whether such differences depended on biophysical setting. Specifically, we examined deviations between lidar- and Landsat-based biomass mapping methods across scales and ecoregions using a measure of error (normalized root mean square deviation), a measure of the unsystematic deviations, or noise (Pearson correlation coefficient), and two measures related to systematic deviations, or biases (intercept and slope of a regression between the two sets of predictions).

**Results:**

Compared to forest inventory data (0.81-ha aggregate-level), lidar and Landsat-based mean biomass predictions exhibited similar performance, though lidar predictions exhibited less normalized root mean square deviation than Landsat when compared with the reference plot data. Across aggregate-levels, the intercepts and slopes of regression equations describing the relationships between lidar- and Landsat-based biomass predictions stabilized (i.e., little additional change with increasing area of aggregates) at aggregate-levels between 10 and 100 ha, suggesting a consistent relationship between the two maps at landscape-scales. Differences between lidar- and Landsat-based biomass maps varied as a function of forest canopy heterogeneity and composition, with systematic deviations (regression intercepts) increasing with mean canopy cover and hardwood proportion within forests and correlations decreasing with hardwood proportion.

**Conclusions:**

Deviations between lidar- and Landsat-based maps indicated that satellite-based approaches may represent general gradients in forest biomass. Ecoregion impacted deviations between lidar and Landsat biomass maps, highlighting the importance of biophysical setting in determining biomass map performance across aggregate scales. Therefore, regardless of the source of remote sensing (e.g., Landsat vs. lidar), factors affecting the measurement and prediction of forest biomass, such as species composition, need to be taken into account whether one is estimating biomass at the plot, stand, or landscape scale.

**Electronic supplementary material:**

The online version of this article (10.1186/s13021-018-0104-6) contains supplementary material, which is available to authorized users.

## Background

Satellite-based maps of forest structure are widely used to understand landscape patterns in vegetation characteristics, to support natural resource planning, and to facilitate carbon mapping and monitoring efforts [1]. Satellite-based maps of aboveground forest biomass upon which carbon monitoring systems are often based have become common due to the temporally deep and spatially complete nature of the data. For example, 30-m spectral reflectance data from the Landsat satellite program provides the capacity for wall-to-wall vegetation mapping from 1984 to present [2, 3]. Despite known limitations, such as the saturation in the relationship between Landsat time series (LTS) data and forest basal area or biomass [4], LTS data form the basis of multi-decadal landscape-, regional-, and continental-scale monitoring of land cover and vegetation attributes [5–7]. However, LTS-based maps can be highly uncertain for individual pixels (i.e., 900 m^2^), with accuracy and precision varying both by response variable and region [1, 8–10]. In contrast, map performance can improve as the geospatial footprint is increased when aggregating data, such as has been seen with imputed forest attribute mapping [2, 11, 12]. Thus, a key challenge for the mapping of carbon stocks, such as aboveground live forest biomass (AGB; Mg ha^−1^), is identifying the minimum appropriate area of aggregation, a task made challenging by the paucity of dense vegetation plot networks.

Plots are sparse and therefore limit the statistical and spatial analyses possible. Fortunately, recent high-resolution AGB maps in many forest landscapes provide opportunities for multi-scale uncertainty assessments. High-resolution, active remote sensing technologies, such as aerial light detection and ranging (lidar), offer an alternative to moderate-resolution, passive sensors, like Landsat Thematic Mapper and Enhanced Thematic Mapper Plus. Lidar-derived AGB maps can provide improved accuracy in mapping relative to satellite-based multispectral sensors [13]. However, current lidar datasets are not spatially extensive, temporally dense, or historically deep, limiting their capacity to form the basis of regional monitoring programs. Nevertheless, they provide valuable information for understanding and correcting prediction errors inherent in existing LTS-based map products. For example, comparisons of lidar and satellite-based AGB maps in Maryland, USA, indicated that local discrepancies in predictions (i.e., differences between mapped aboveground live biomass at some aggregate-level) were common, generally leading to a net underestimate of biomass in satellite-based maps compared to lidar-based maps, with these differences persisting even when comparing averages across the entire study region [14]. However, it is not known how common such patterns are across other forest landscapes.

Even within a single landscape, uncertainty in the relationship between remote sensing and forest biomass should be expected to vary, likely as a function of vegetation gradients themselves [9]. Mapping methods that incorporate both remote sensing and environmental data implicitly account for some of this variation [12, 15], allowing environmental data to modify the relationship between remote sensing and plot data. High resolution, direct measurement of vegetation height using lidar, and the close relationship of these data to vegetation structure [16], can provide more accurate aboveground biomass maps compared to optical remote sensing [17]. Similarities amongst regression models relating lidar height metrics to AGB across broad regions highlight the relative insensitivity of AGB predictions to structural and compositional variation [18, 19]. However, regional variation in allometric equations has a substantial impact on model development and performance [20] and might result in variation in remote sensing-derived aboveground biomass map accuracy.

In this work, we compared lidar-based and LTS-based predictions of mean AGB at scales from sub-hectare to landscapes (10 s to 1000 s ha) to assess factors contributing to local discrepancies between biomass map products. The degree to which LTS and lidar maps converge (i.e., become increasingly similar as aggregate scale increases) is likely to be a function of the ecosystem types being examined. Such information will help to define appropriate scales of aggregation and landscape-level correlates with varying performance for LTS-based AGB mapping products—essential for its continued use by land managers, policy makers, and scientists alike. As a result, providing map users with a multi-scale comparison of differing products and assessing how performance varies as a function of the vegetation characteristics themselves may provide valuable guidance to planners and decision-makers assessing the current status of forest carbon stocks. We address the following questions: (1) at what aggregate-level do lidar and LTS based predictions of mean aboveground biomass become equivalent, if at all? (2) Do unsystematic (i.e., noise) and systematic deviations (i.e., biases) change as biophysical setting (e.g., percentage of the landscape classified as forest [percent forested], forest canopy cover, and percent hardwood) changes?

## Methods

### Study area

**Fig. 1:**
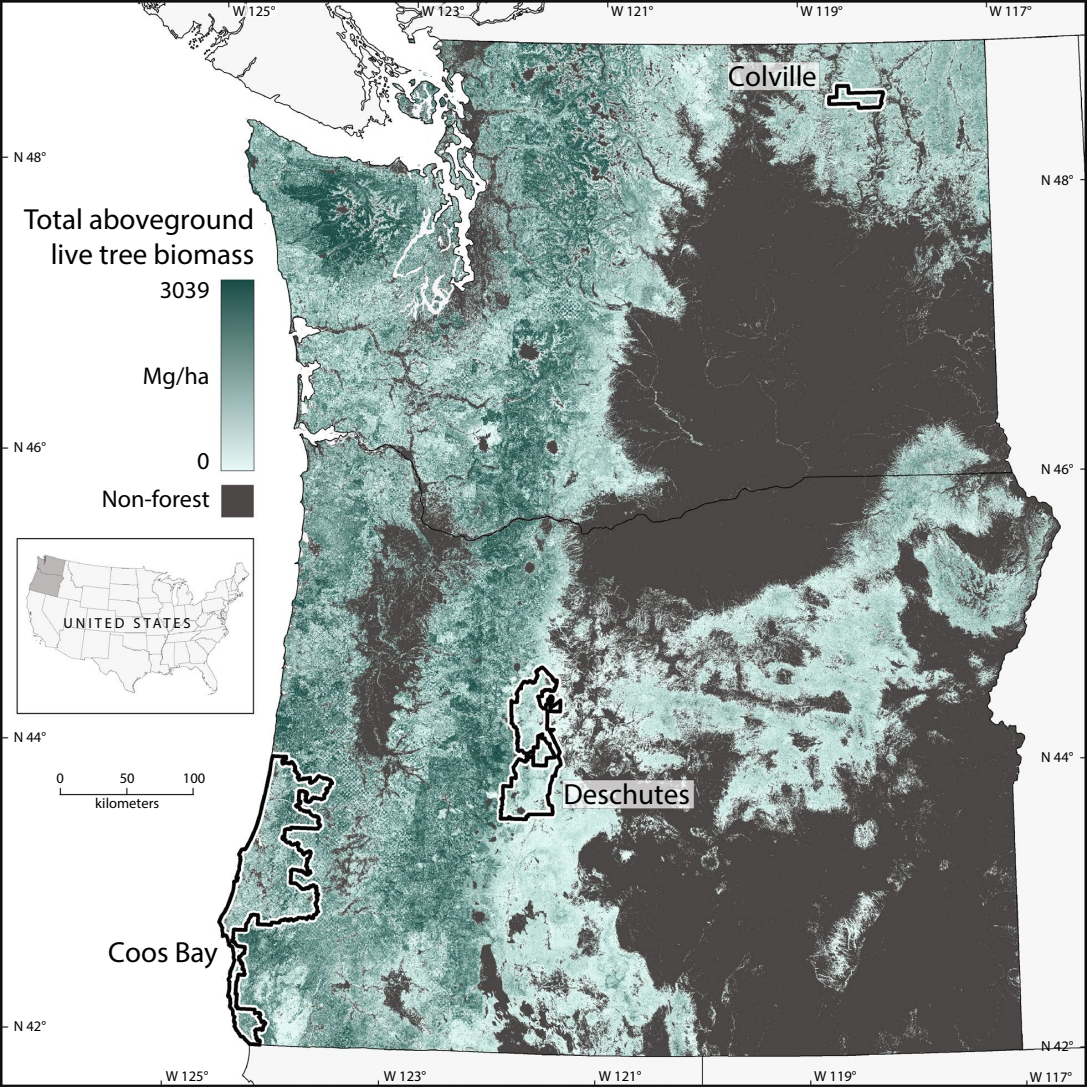
Study region map of total aboveground forest biomass based on LTS and the gradient nearest neighbor (GNN) imputation methodology

This study focuses on three study regions—Coos Bay, Colville, and Deschutes—located in Oregon and Washington, USA for which a single acquisition of aerial lidar data was available (Fig. 1, Table 1). Study regions range in size from 347 to 4609 km^2^, encompassing portions of three to eight different level 4 ecoregions [21] that range in size from 10 to 2369 km^2^, with 0.7–37.8% of each ecoregion incorporated in the current analysis (Table 1). Therefore, while the full extent of each study region was examined in this study, a minority of area encompassing each ecoregion that intersects a given study region was included. The study regions have diverse patterns of land ownership and management, and represent broad gradients in climate and vegetation based on parameter elevation relationships on independent slopes model (PRISM) climate data [22] and forest inventory and analysis (FIA) data [23], respectively (Additional file 1: Figure S1). Coos Bay is a warm, wet region and is comprised of forests with a greater proportion hardwood and forests with greater mean forest canopy cover compared to the other two study regions. Deschutes is cooler and drier than Coos Bay, with little hardwood basal area and with mean forest canopy cover ranging from 10 to 70% across ecoregions (Additional file 1: Figure S1). Colville has similar temperature, but less precipitation than Deschutes, and has similar mean forest cover and hardwood proportion. All three regions exhibit high variation in percentage of land classified as forest based on the National Gap Analysis Project [24]. The variation in forest landscapes within and across study regions represents much of the range of AGB across Oregon and Washington (Table 1).

**Table 1:** Description of the study regions and ecoregions (area within study regions only)

Study region	Ecoregions	Lidar year	Forest area within ecoregion		Mean GNN biomass (Mg ha^−1^)	Mean lidar biomass (Mg ha^−1^)
			(km^2^)	(%)		
Coos Bay		2008–2009	4609		194.6	244.1
	Coastal Lowlands		464	13.6	100.9	141.6
	Coastal Uplands		758	11.1	146.9	199.7
	Coastal Siskiyous		203	9.2	216.9	191.7
	Inland Siskiyous		78	1.2	180.8	210.3
	Mid-Coastal Sedimentary		2369	24.5	235.2	302.1
	Northern Franciscan Redwood Forest		28	0.7	333.7	361.7
	Southern Oregon Coastal Mountains		670	37.8	178.3	195.4
	Umpqua Interior Foothills		39	1.7	128.5	187.5
Colville		2008	347		86.1	111.1
	High Northern Rockies		10	2.3	67.6	57.4
	Okanogan-Colville Xeric Foothills		73	1.0	55.5	83.4
	Okanogan Highland Dry Forest		265	5.4	97.9	123.4
Deschutes		2009–2010	2572		80.2	82.2
	Cascade Crest Montane Forest		60	1.1	12.6	13.9
	Cascade Subalpine/Alpine		508	25.5	114.1	91.9
	Grand Fir Mixed Forest		78	3.1	88.1	58.1
	Ponderosa Pine/Bitterbrush Woodland		74	2.6	125.7	108.1
	Pumice Plateau		1020	9.3	70.4	84.2
	Pumice Plateau Basins		515	31.0	90.5	100.5
	Deschutes River Valley		317	7.8	47.5	51.7

Further details on lidar acquisitions in Additional file 1

### Total aboveground biomass maps

This project used lidar- and LTS-based mean AGB maps for each study region collected from 2008 to 2010, as determined by the year during which lidar data were acquired (Table 1). Therefore, LTS- and lidar-based AGB maps were compared for the same year within a study region. Regardless of data source (i.e., lidar vs. LTS), plot data with individual tree measurements were available, with species and tree diameter. We used the component ratio method for predicting aboveground live tree biomass [25, 26], summed tree-level predictions to plot-level, and rescaled AGB to the per unit area level (i.e., Mg ha^−1^). While we briefly describe the LTS- and lidar-based modeling methods below, they are described in greater detail in [3].

### LTS-based AGB mapping

LTS-based maps were developed independently for broad modeling regions roughly equivalent to level 3 ecoregions [21] using the gradient nearest neighbor (GNN) imputation method. Level 3 ecoregions are broader in scope than the level 4 ecoregion or the study regions described in Table 1, and therefore incorporate FIA plot data from a wider selection of forest ecosystems. While the use of FIA plots from beyond the bounds of the study regions likely provides more information on forest conditions with which to develop AGB maps, there are likely still limits to our capacity to map extreme conditions, such as very high biomass forests. GNN imputation is a flexible, non-parametric modeling and mapping methodology that relates measured forest attribute data from inventory plots to geospatial information, including LTS data and abiotic environmental data [12, 15, 27]. Plot data (basal area for species by size class groupings) and geospatial predictors (both LTS and environmental variables from regional or national geospatial datasets) were extracted for each plot for the year that the plot was measured. Across eight contiguous modeling regions in Oregon and Washington, sample sizes for GNN modeling ranged from 2024 to 5587 plots. The inventory plots covered a sub-sample of a roughly 90-m by 90-m footprint (9 Landsat pixels) [28] for which all geospatial predictors were extracted and averaged for the purposes of modeling. A canonical correspondence analysis (CCA) [29] was performed to define a multivariate gradient space. For each 30-m pixel in the study area, AGB from the plot identified as the nearest neighbor (i.e., minimum distance) in gradient space was imputed to that pixel. Since all geospatial predictors were available from 1984 to 2012 (duration of Landsat 5), annual AGB maps were generated and the year most closely matching the lidar data acquisition dates (Table 1) was used for further comparisons. Further details regarding data and modeling can be found in the Additional file 1 and [3]. Hereafter, we refer to the AGB maps produced using GNN as LTS-based data or maps.

### Lidar-based AGB mapping

Lidar-based maps were developed for each study region independently using a multiple linear regression framework [3]. The lidar acquisition and model fitting process is described in greater detail in Additional file 1. Each lidar acquisition has an associated plot network (*n* = 893, 157, and 303 for Coos Bay, Colville, and Deschutes, respectively) and characteristics of the lidar acquisition for calibrating AGB models. Note that the lidar plots were not the same as the forest inventory plots used for the LTS-based AGB mapping. Lidar field plots were generally smaller in area with fixed radius designs whereas FIA plots were larger with a nested design, though both sets of plots provided individual tree data to which allometric equations could be applied and scaled to a per-hectare measure of AGB (see Additional file 1 for details). In both cases, the component ratio method was used to calculate AGB from plot data [25]. A single regression model and AGB map was produced for each study region. Generally, measures of canopy height (e.g., 90th percentile height), variability in the height of returns (e.g., coefficient of variation in height), and/or the vertical distribution of lidar returns (e.g., proportion of lidar returns above 2 m height) were the strongest covariates for most regions and models performed well for the regions within which they were developed (*R*^2^ ≥ 0.69) (Additional file 1) [3]. Maps were aggregated to 30-m resolution to match LTS-based AGB maps.

### Map comparisons

We compared lidar-based and LTS-based mean AGB (Mg ha^−1^) maps across aggregate-levels from 90-m (0.81 ha) to 7290-m (5314.41 ha). Because annual LTS-based maps were generated for 1984–2012, we used LTS-based maps for the same year as the lidar acquisition in each study region (Table 1) to minimize differences due to the timing of remote sensing. The minimum 90-m aggregate-level roughly matches the footprint represented by FIA plots [23] used for assessment of the two maps and to parameterize the GNN models. The 90-m aggregate-level helps to minimize potential edge effects and uncertainties associated with the underlying lidar and LTS data [30]. The 90-m aggregate-level also avoids errors associated with comparing map data at spatial resolutions finer than the input data [28]. At the 90-m aggregate-level (i.e., plot-level), we compared (1) biomass predictions (lidar or LTS) with FIA field observations as reference data and (2) LTS-based biomass predictions with the lidar biomass predictions as reference data. LTS-based AGB accuracy assessments with FIA observations avoided comparing the field data with a nearest neighbor imputation using the same plot through a modified leave-one-out validation procedure [15].

At all aggregate-levels (≥ 90-m), we compared mean LTS-based AGB predictions with mean lidar AGB predictions as reference data. For each aggregate, the mean pixel-level AGB was calculated. We performed comparisons separately for each level 4 ecoregion within a study region (Table 1) to assess differences in performance across disparate forest ecosystems. Performing comparisons at the scale of individual ecoregions acts to stratify the data within a study region by biophysical setting. However, given that a minority of the total area of a given ecoregion is represented in our three study regions (Table 1), comparisons may not be representative of the ecoregion as a whole. While we used ecoregion to stratify the study region based on biophysical setting, one might also stratify based on any variable of interest, such as elevation, climate, or topography. For LTS-based AGB maps, pixel values for non-forest land based on 1999–2001 land cover data from the National Gap Analysis Project [24] were set to 0.0 Mg ha^−1^ whereas lidar-based maps can include non-zero predictions for those same locations. Because comparisons on lidar- and LTS-based biomass estimates in non-forested lands would be confounded with this masking process, we elected to compare products only in the forested portion of the landscapes under consideration. Analyses based on aggregation across all pixels produced qualitatively similar results as the same analyses using only forested pixels, possibly because lidar acquisitions were focused on forest lands, minimizing the effects of non-forest lands on estimates at coarser aggregate levels.

For a given aggregate-level, we tallied the number of pixels within each aggregate that fell within the area of interest (i.e., forest lands in a study region or ecoregion). If fewer than 20% of pixels within an aggregate were forested, based on GAP land cover data [24], that aggregate was excluded from our analysis. Inclusion thresholds as high as 90% produced qualitatively similar results, so we chose 20% to maximize the number of aggregates retained, allowing for examination of broader aggregate-levels. To minimize storage and computational needs, we aggregated data to every 4th resolution (i.e., 3-by-3 pixels, 7-by-7 pixels, 11-by-11 pixels etc.), excluding pixels intersecting with the edge of a study region or ecoregion. To avoid major impacts of edge effects on our analysis, only aggregates for which at least 75% of their area was encompassed in the associated study region or ecoregion were retained for analysis. We defined the maximum aggregate-level for a given ecoregion as the maximum aggregate-level at which 30 aggregates still meet the above criteria. Thus, aggregate size ranged from 0.81 to 5314.41 ha, depending on the size of the ecoregion of interest (Table 1). The resulting aggregated datasets allow for comparisons between lidar- and LTS-based AGB maps across stand- to landscape-scales for entire individual ecoregions, as well as study regions. For study regions, means and 95% confidence intervals for ecoregion-level performance metrics were calculated at each aggregate level.

### Performance metrics

No single performance metric will be sufficient for examining when and how deviations between two maps or a map and field observations arise. For example, performance of forest carbon maps for conterminous United States were assessed at multiple scales using agreement coefficients, Kolmogorov–Smirnov statistics, and reduced major axis regression at pixel-levels ranging from 25 to 200 km [11]. In this research, we performed comparisons of lidar- and LTS-based AGB maps using a suite of performance metrics selected to characterize different components of the deviations between the two datasets. Specifically, we calculated the normalized root mean square deviation, the Pearson correlation coefficient, and the intercept and slope terms from a linear regression to characterize the magnitude and type of deviation between LTS- and lidar-based AGB maps.

Similar to Huang et al. [14], overall deviations between the two AGB maps were assessed using the normalized root mean squared deviation $\left( {{\text{NRMSD}}\, = \sqrt {\sum\nolimits_{i = 1}^{n} {n^{ - 1} \left( {x_{i} - y_{i} } \right)^{2} } } } \right),$ where *y*_*i*_ is the lidar-based prediction and *x*_*i*_ is the LTS-based prediction. In this context, the lidar predictions are taken to be the reference dataset for the purposes of NRMSD, even though both maps contain error. We used NRMSD instead of root mean square deviation so that error across regions and ecoregions with differing magnitudes of observed AGB could be directly compared. To explore unsystematic deviations (i.e., noise), we used the Pearson correlation coefficient, defined as the linear correlation between lidar- and LTS-based AGB predictions, with values near 1 indicating little difference between the two datasets associated with noise. To explore systematic deviations (i.e., bias), we characterized the relationship between the lidar- and LTS-based AGB maps using linear regression. Specifically, we performed linear regressions with the LTS-based AGB as the predictor variable and the lidar-based AGB as the response variable. Intercept parameters described the lidar-based AGB with LTS-based AGB equals zero, where intercepts greater than or less than 0 indicated that the lidar-based AGB were greater than or less than LTS-based AGB, respectively. Slope parameters described the change in lidar-based AGB with an increase in LTS-based AGB, where values different from 1 indicated that differences between lidar-based and LTS-based AGB varies as a function of AGB itself.

### Relationship between deviations and forest structure

**Fig. 2:**
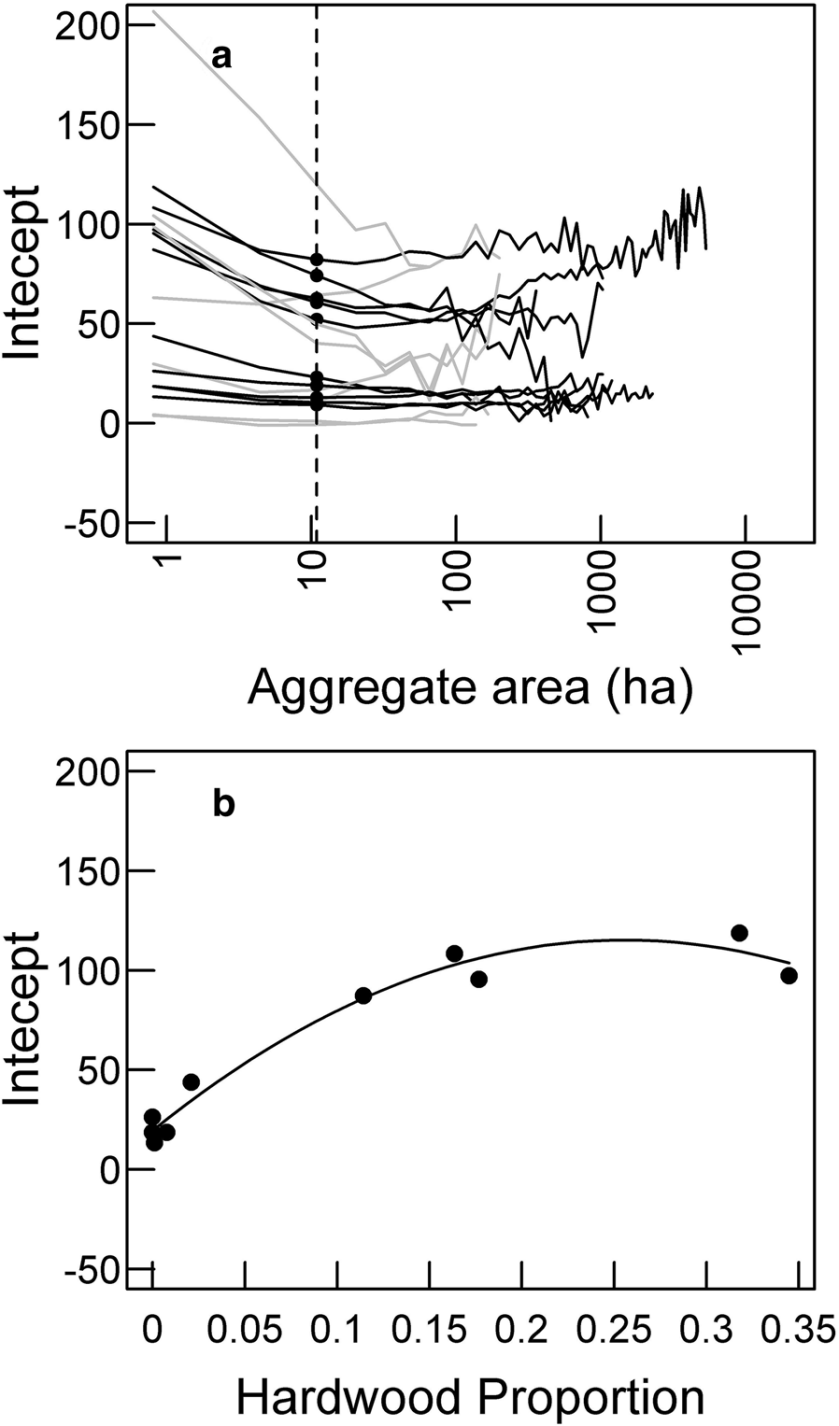
An example of assessing the relationship between a performance metric (e.g., the intercept) and vegetation characteristics (e.g., hardwood proportion) across ecoregions. **a** For large ecoregions (i.e., at least 30 individual 313-ha aggregates could be sampled) in all study areas, a given performance metric is calculated across a range of aggregate-levels (black solid lines). At a given aggregate-level (vertical dashed line), the performance metric is extracted across ecoregions (black points). Smaller ecoregions (gray lines) were excluded. **b** The performance metrics for those points (black points) are regressed against a vegetation characteristic (solid line)

To determine whether deviations between lidar and LTS AGB maps vary with forest structure, we regressed performance metrics for different ecoregions at aggregate-levels from 0.81 ha (3-by-3 pixels) to 313 ha (59-by-59 pixels) on mean vegetation characteristics calculated from the FIA plots located within a given ecoregion (Fig. 2). Because the inclusion of smaller ecoregions tended to create outliers in the relationships between mean vegetation characteristics and performance metrics, we limited this analysis to the 10 ecoregions distributed across the three study regions for which at least 30 individual 313-ha aggregates could be generated: Cascade Subalpine/Alpine, Coastal Lowlands, Coastal Uplands, Coastal Siskyous, Deschutes River Valley, Mid-Coastal Sedimentary, Okanogan Highland Dry Forest, Pumice Plateau, Pumice Plateau Basins, and Southern Oregon Coastal Mountains. This ensured that changes in relationships with scale did not represent changes in the sampling pool as smaller ecoregions were excluded at larger aggregate-levels. Performance metrics for a given aggregate-level were calculated for the 10 large ecoregions within the three study regions (Fig. 2a). For these large ecoregions, we calculated several mean vegetation characteristics from FIA data within each ecoregion: percent of landscape classified as forest (i.e., percent forested), mean canopy cover of forested areas, and hardwood proportion of forested areas. We selected these characteristics to represent spatial heterogeneity in forest distribution as well as structural and compositional variation within forested portions of the landscape.

To understand some of the drivers of interregional variation in the performance metrics, we regressed each metric against vegetation characteristics for the ecoregions (Fig. 2b). Regressions of performance metrics on vegetation characteristics utilized linear regressions for NRMSD, intercept and slope and beta regression for correlation. We examined explanatory capacity with the coefficient of determination (*R*^2^) from linear regressions and pseudo-*R*^2^ from beta regressions. We performed linear regressions and beta regressions using the lm and betareg (from betareg package, version 3.1-0) [31] functions in the R statistical programming language version 3.3.2 [32].

## Results

### Comparing AGB maps to field measurements

**Fig. 3:**
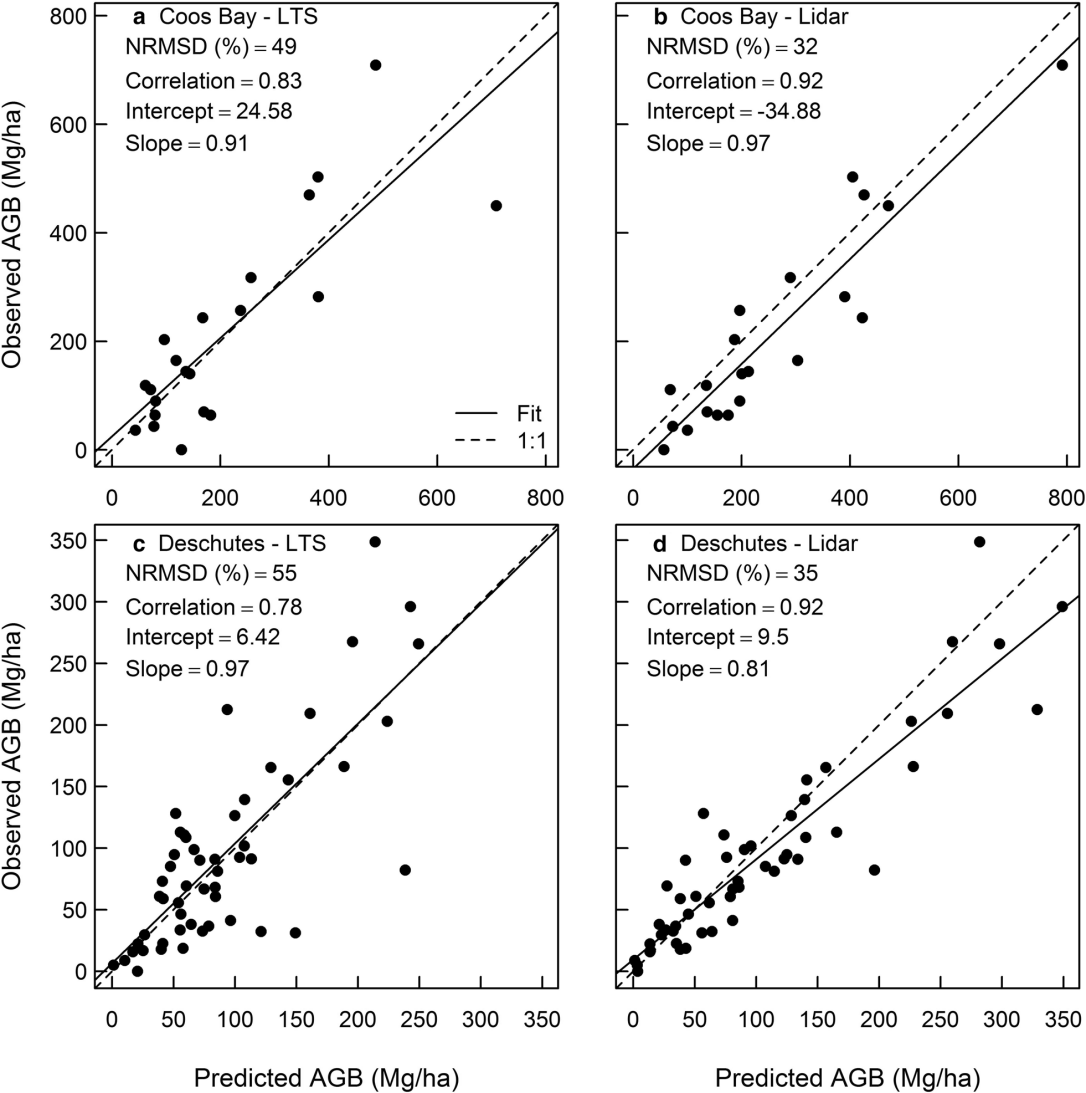
Predicted vs. observed aboveground biomass (AGB) for FIA plots in the Coos Bay (**a**, **b**) and Deschutes (**c**, **d**) study regions based on LTS (**a**, **c**) and lidar (**b**, **d**) with associated model performance statistics. Dashed line indicates 1:1 line and solid line indicates a regression (observed AGB = f[LTS or lidar AGB]) based on the maximum likelihood estimate. For each comparison, we provide normalized root mean square deviation (NRMSD), Pearson correlation coefficient, and the linear regression parameters (intercept and slope)

Two of the study regions were large enough to contain numerous FIA plots: Coos Bay (*n* = 21) and Deschutes (*n* = 52). In both study regions, lidar-based AGB predictions performed better than LTS-based predictions, exhibiting lesser NRMSD and greater Pearson correlation coefficients (Fig. 3). In Coos Bay, lidar-based estimates were more biased than LTS-based estimates, tending to overpredict AGB. In Deschutes, biases in LTS and lidar estimates were similar, though lidar tended to overpredict for high AGB. Additionally, saturation in the biomass predictions was observed for LTS-based AGB in Deschutes, but less so in Coos Bay and not for the lidar-based AGB predictions. Thus, lidar-based AGB maps compared to LTS-based maps exhibited less error and less noise, but some evidence of more bias when predicting forest inventory plot data for Coos Bay and Deschutes regions.

### Comparing lidar- and LTS-based AGB

**Fig. 4:**
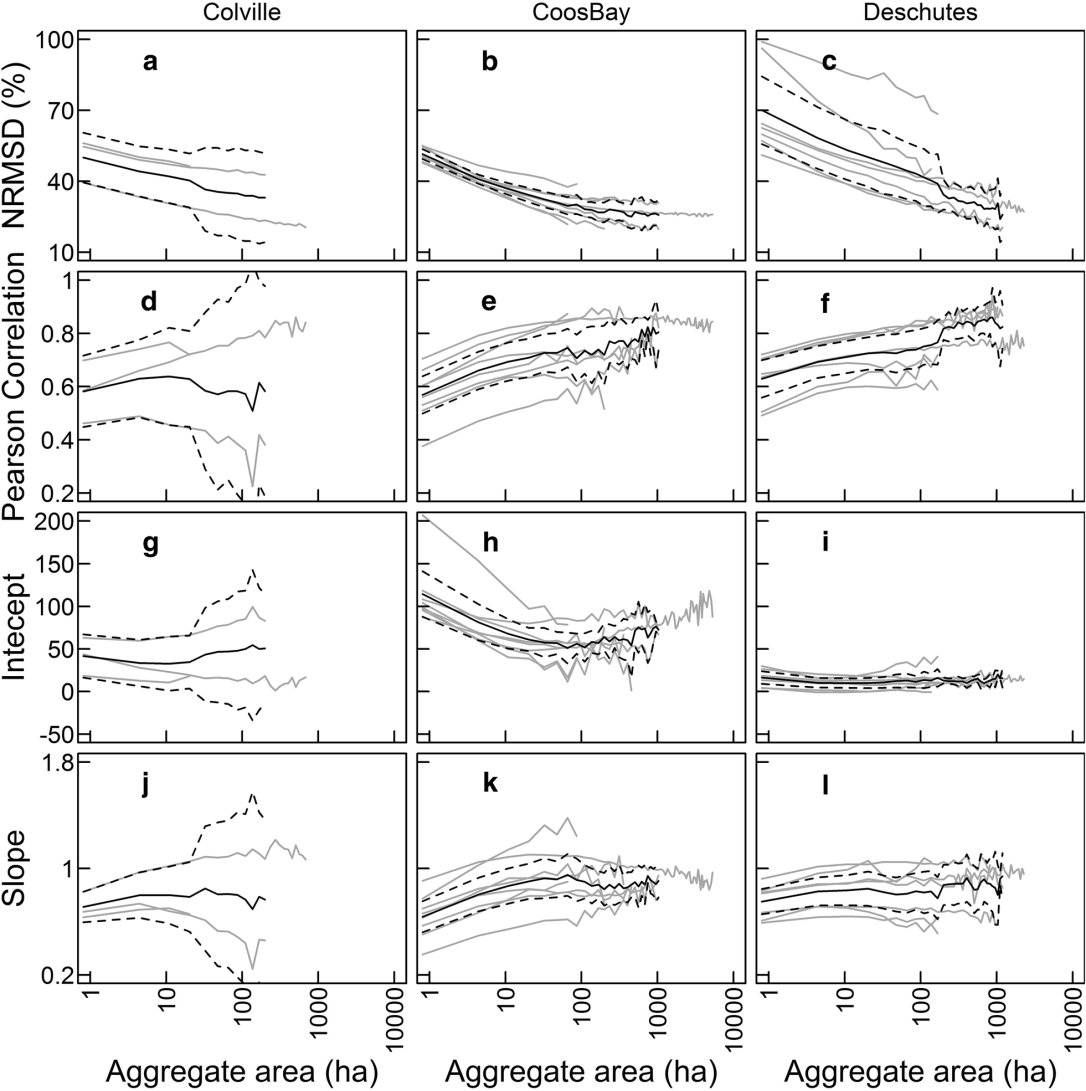
Means with confidence intervals (black solid and dashed lines, respectively) and observed ecoregion-level (gray lines) performance metrics summarizing deviations between LTS-based and lidar-based AGB mapping for ecoregions within study regions at different aggregate-levels. **a**–**c** NRMSD = normalized root mean square deviation; **d**–**f** Pearson correlation coefficients; **g**, **i** intercept and **j**–**l** slope are simple linear regression parameters with lidar-based AGB as the response variable and LTS-based AGB as the predictor variable

Within study regions and ecoregions, NRMSD declined across aggregate-levels, often by as much as half from 0.81 to 100 ha (Fig. 4a–c). Pearson correlation coefficients increased by 0.1–0.2 across finer aggregate levels (0.81–100 ha), followed by little or not increase within most ecoregions at coarse aggregate-levels (Fig. 4d–f). Between 10 and 1000 ha, we observed stabilization in simple linear regression parameters indicating positive intercept terms (Fig. 4g–i) and slopes < 1.0, though 95% confidence intervals included 1.0 for most aggregate-levels in the Colville study region, for aggregate-levels > 10 ha in the Coos Bay study region, and for aggregate-levels > 166 ha (Fig. 4j–l). These results indicated that lidar-based AGB was greater than LTS-based AGB at lesser values of AGB, with that difference diminishing or changing direction for forests with greater AGB values. Noise in performance metrics when aggregate area was large was caused by decline number of aggregates used to calculate ecoregion-level performance and relatively low numbers of ecoregions with at least 30 aggregates in a given study region (i.e., as aggregate-level increased, fewer ecoregions were incorporated in the analysis).

**Fig. 5:**
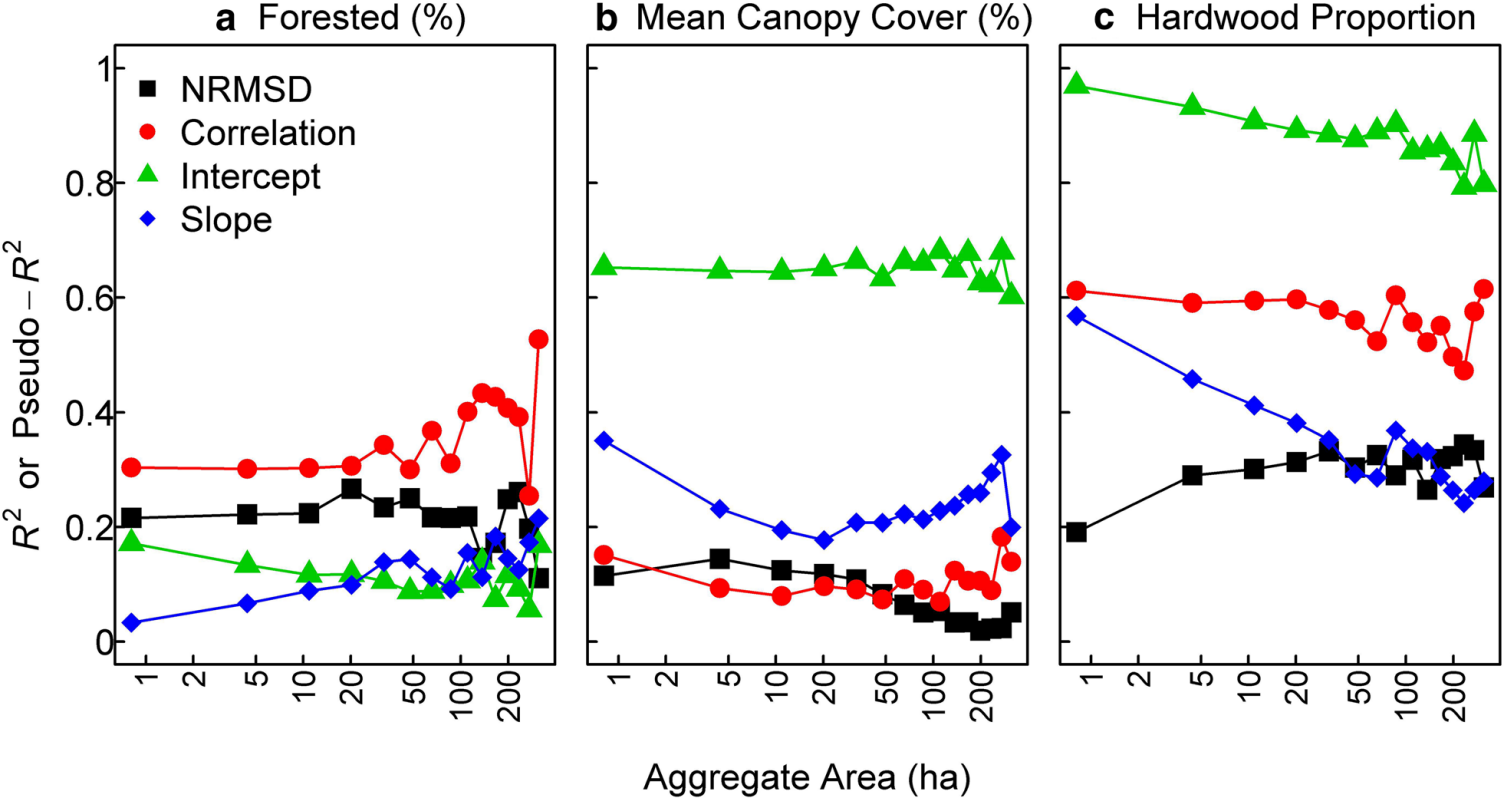
Capacity of **a** percent area classified as forest, **b** mean tree cover within forests, and **c** proportion of forest basal area comprised of hardwoods to explain performance metrics for ecoregions at differing aggregate-levels. Explanatory capacity is represented by coefficient of determination (*R*^2^) from linear regression models and pseudo-*R*^2^ from beta regression models with the vegetation characteristic as the predictor and the performance metric as the response

Using linear regression, we examined how the performance metrics at the ecoregion-level varied as a function of vegetation characteristics (Fig. 5). By examining the *R*^2^ or pseudo-*R*^2^ for models with a main and quadratic effect, we were able to identify which performance metrics varied regionally as a function of vegetation characteristics: large *R*^2^ or pseudo-*R*^2^ indicated that ecoregion-level performance metrics varied as a function of vegetation characteristics. Percent forested was weakly related (*R*^2^ or pseudo-*R*^2^ > 0.35) to Pearson correlation coefficient (most aggregate-levels) (Fig. 5a). Mean canopy cover within forested landscapes was related (*R*^2^ > 0.50) to the additive deviations between the two AGB maps (intercept) at all aggregate-levels (Fig. 5b). Moderate to strong relationships (*R*^2^ or pseudo-*R*^2^ > 0.50) were observed between hardwood proportion and the intercept term and Pearson correlation coefficient at all aggregate-levels and the slope term at aggregate-levels < 5 ha (Fig. 5c).

**Fig. 6:**
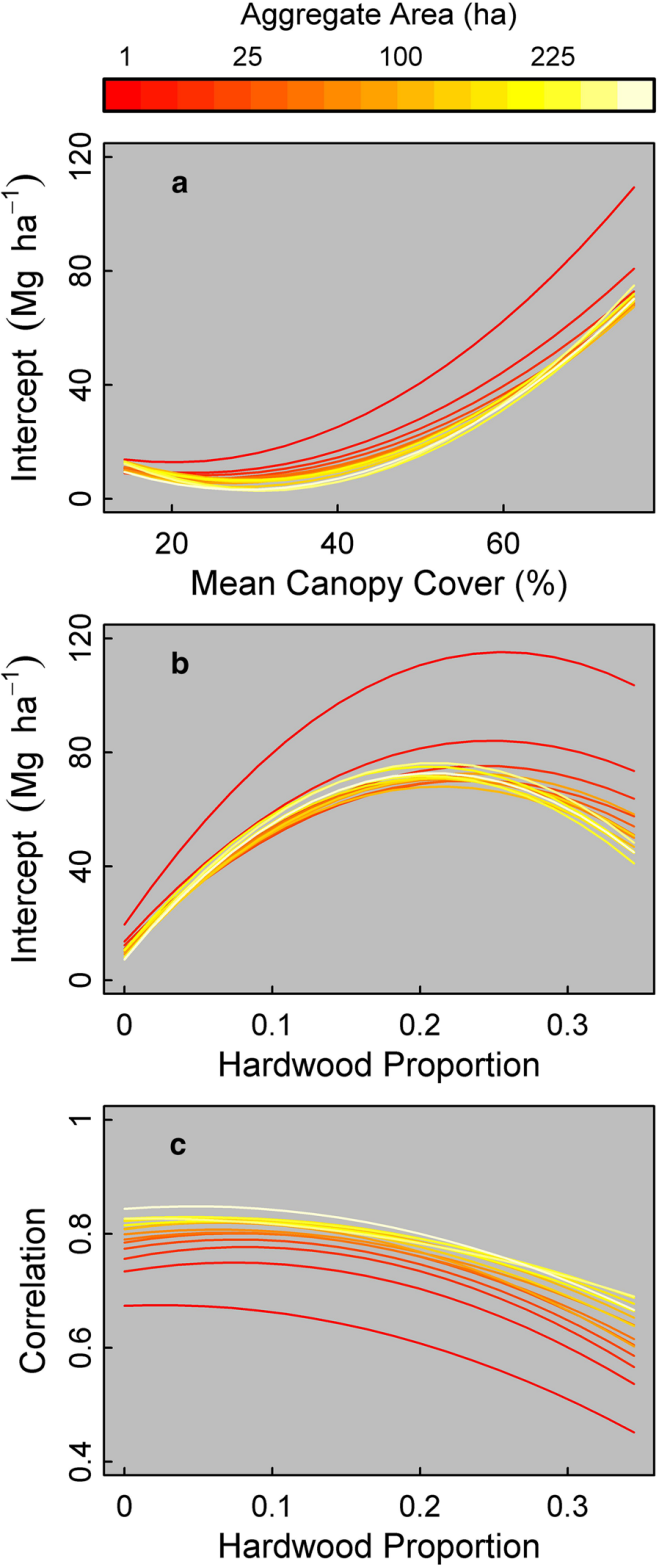
Fitted relationships between the ecoregion-level (**a**, **b**) intercept and **c** Pearson correlation coefficient from a simple linear regression models with lidar-based AGB as the response variable and LTS-based AGB and vegetation characteristics as the predictors: **a** mean forest canopy cover, and **b**, **c** hardwood proportion. Each line is the regression fit at a given aggregate-level, ranging from approximately 0.81 ha (red) to hundreds of hectares (yellow). Only the 10 largest ecoregions were included

Due to the relatively high *R*^2^ or pseudo-*R*^2^ values (Fig. 5), we further examined the relationship of the linear regression intercepts with mean forest canopy cover and hardwood proportion as well as the relationship of Pearson correlation coefficient with hardwood proportion. The regression intercepts between the two AGB maps increased with mean forest canopy cover and hardwood proportion (Fig. 6a, b). Pearson correlation coefficients between the two AGB maps decreased with hardwood proportion (Fig. 6c). The observed increases in the regression intercept with mean canopy cover and hardwood proportion and decreases in correlation with hardwood proportion were consistent across aggregate-levels, indicating a consistent set of differences between lidar- and LTS-based AGB maps related to vegetation characteristics. Overall, these results show that forest canopy characteristics impacted both systematic (intercept) and unsystematic (Pearson correlation) deviations between the two datasets.

## Discussion

Stand- to landscape-level predictions of AGB based on lidar and LTS data differed in a consistent and predictable fashion across regions and aggregate-levels, although important differences emerged as a function of biophysical setting, as represented by ecoregion-level mean vegetation characteristics. Landscape-scale vegetation characteristics were important in explaining differences in mapping discrepancies among ecoregions (Fig. 5). In particular, we observed strong relationships between hardwood proportion and the regression intercepts and correlation coefficients, indicating that ecoregions where hardwoods and conifers both contributed substantially to AGB, the predictions exhibited strong systematic differences (i.e., increasing intercepts) and increasing imprecision (i.e., reduced correlation) (Fig. 6b, c). Previous comparisons of regionally-derived lidar AGB prediction performance across five sites in the Pacific Northwest USA indicated that mean AGB was predicted well at each site, but that hardwood basal area, which is highly correlated with biomass, was poorly predicted at two of the sites [19]. Studies in California have indicated that lidar predictions are improved through stratification procedures based on vegetation classification [33, 34]. Thus, a single model applied to all lands may ignore the complex contributions of different components of the ecosystem, such as differing tree species, to AGB patterns, as has been noted for lidar-based tree basal area models for lower and upper canopy trees [35]. A possible solution would be to fit lidar-based AGB models for each ecoregion separately to account for regional variation in tree species composition (e.g., [33]). Alternatively, utilizing existing maps of tree species composition could be used as predictor variables in lidar-based AGB modeling. Finally, fusion of lidar-based and Landsat-based biomass maps could provide the basis for addressing known limitations in each mapping methodology (e.g., [36]). However, uncertainties in the maps of species composition used in these proposed approaches will certainly impact resulting maps and should be considered carefully.

Discrepancies between lidar- and LTS-based AGB maps related to tree species composition might also be explained by uncertainties in allometric equations impacting modeling, and thus predictions [20]. For example, if uncertainties and errors in allometric equations differ between hardwoods and conifers or between closed canopy forest tree species and woodland tree species [37], then spatial variation in the hardwood proportion or forest cover, respectively, would differentially affect model performance. A recent assessment of uncertainties in FIA allometric equations indicated that the greatest uncertainties in current methods may be associated with woodland ecosystems of the western United States [38] where mean forest canopy cover would be low. However, our results were inconsistent with such an explanation, as ecoregions with low canopy cover (< 30%) exhibited lesser intercepts (Fig. 6a). While our results were not consistent with allometric uncertainties determining differences between the lidar- and LTS-based methods, our results appear to caution against naïve prediction of biomass without accounting for major differences in vegetation characteristics.

Despite substantial geographic, ecological, and environmental differences between study regions and ecoregions examined in this study (Additional file 1: Figure S1), consistent patterns of increasing agreement (i.e., decreasing NRMSD) at coarser aggregate-levels emerged (Fig. 4). In part, increasing agreement with scale likely reflects the tendency of both methods to produce reasonable predictions of mean AGB over large areas. Furthermore, under-prediction of high AGB by Landsat-based predictions associated with spectral saturation [4, 39] are less noticeable when averaged with other predictions at broader aggregate levels. Previously reported poor performance of LTS-based maps [14] are, at least in part, due to scaling issues in model validation. For example, one such assessment found poor performance of the LTS-based maps in the Pacific Northwest, but this was based on field plots capturing structure across areas < 0.1 ha that resulted in a scaling mismatch [28]. Given increasing Pearson correlation coefficients and the intercept and slope parameters from regressions between lidar- and LTS- based AGB approaching 0 and 1, respectively, our results highlight declining unsystematic (i.e., noise) and systematic (i.e., bias) deviations between lidar- and LTS-based AGB maps as we transition from plot-level (0.81 ha) to coarser aggregate-levels (> 10–100 ha).

At aggregate-levels of 10–1000 ha, relatively stable relationships between the lidar- and LTS-based predictions, represented in this paper by intercepts and slopes from regression analyses (Fig. 4), indicated that the two types of AGB maps represent similar gradients from low to high biomass forests needed by natural resources professionals. Positive intercepts and slopes < 1 (Fig. 4g–l) tend to indicate that LTS under-predicts AGB relative to lidar most strongly in low AGB landscapes, not high AGB landscapes as might be expected if saturation impacted landscape-level estimates. Given the commonly cited impact of saturation in the relationship between spectral data and AGB [4] and the apparent saturation observed in LTS-based predictions for FIA plots in the Coos Bay study region (Fig. 3c), it was surprising to find that comparisons between lidar- and LTS-based AGB at aggregate scales did not indicate a major role of saturation in biasing landscape-level estimates. One possible interpretation of these results is that, at aggregate-levels greater than 100 ha, lidar and LTS can similarly represent landscape gradients in the mean state of these forests from low to high AGB, even though predictions at the scale of individual pixels can differ substantially due to limitations in the remote sensing, such as spectral saturation. Another possible explanation is that the act of aggregating data limits the importance of errors when predicting rare components of the landscape, such as high biomass forests. Stable relationships between the two sets of predictions (i.e., intercepts and slopes) are interesting in that they suggest a similar capacity to identify landscape-scale vegetation patterns. For example, landscape monitoring efforts aimed at assessing the distribution of high vs. low biomass forests might effectively utilize either lidar- or LTS-based products, even though actual biomass predictions will tend to differ.

However, the stability in some performance metrics across aggregate-levels does not indicate that the lidar- and LTS-based predictions are equivalent. For example, the combination of positive intercepts and slopes < 1 (Fig. 4) indicated that lidar-based maps predicted greater AGB than LTS-based maps within low biomass forests, but that this difference declines in magnitude or even changes direction (i.e., LTS-based AGB > lidar-based AGB) as the regression line crosses the 1:1 line at greater AGB values. Still, increases in Pearson correlation coefficients with aggregate area indicated that LTS-based AGB data could be linearly transformed to correct for most of the discrepancy. Linearly transforming, or calibrating, aggregate LTS-based predictions based on lidar predictions would vary among disparate vegetation types, as evidenced by differences in slopes and intercepts between ecoregions.

In this research, we utilized existing lidar-based AGB maps that use only structural metrics from the lidar data, a common practice in many regions. As opposed to the regression methods used when developing our lidar-based AGB maps, the GNN framework implicitly incorporates environmental constraints on vegetation mapping, such as climatic controls on tree species distributions, allowing the relationship between remote sensing and AGB to vary as biophysical setting changes [15] whereas our lidar-based regression analysis did not. As a result, the variation in intercept with hardwood proportion may represent a difference between regression and GNN modeling, rather than a fundamental difference between lidar and LTS-based approaches. This is consistent with the observed improvements in lidar-based biomass mapping when accounting for biophysical setting, such as species composition [33, 34]. Comparative frameworks such as the one utilized in this research offer an opportunity for comparing map products based on different remote sensing and different statistical methods to understand how and why map errors occur, which is a valuable area of future research. We also note that comparisons across a broader set of biophysical settings would allow for the development of a deeper understanding of the drivers of deviations between differing map products, potentially leading to improved modeling procedures.

## Conclusions

Our multiscale comparison of lidar- and Landsat-based AGB predictions provided new insights into how these differing data sources can support forest biomass and carbon management across scales. Deviations between lidar- and Landsat-based maps indicated that these differing approaches represent similar gradients in forest biomass. However, ecoregion impacted these deviations, highlighting the importance of biophysical setting in determining map performance across aggregate scales. Gradients in species composition may need to be incorporated into lidar-based AGB mapping and any calibration of LTS-based AGB that utilizes the lidar-based predictions. Given that map differences depended on vegetation pattern, such as the distribution of plant function types (e.g., hardwood vs. conifer trees) and forest cover, users should be wary of biomass and carbon mapping efforts that do not account for variation in biophysical setting. Finally, these results imply an exciting potential for fusion of lidar- and Landsat-based maps to produce calibrated AGB products that leverage the strengths of multiple remote sensing technologies into a single mapping framework.

## Additional file

**Additional file 1.** Additional details regarding ecoregion descriptions, Landsat-based biomass modeling, and lidar-based biomass modeling.

## Abbreviations

AGB: aboveground biomass
CCA: canonical correspondence analysis
FIA: forest inventory and analysis
GNN: gradient nearest neighbor
LTS: Landsat time series
NRMSD: normalized root mean square deviation
PRISM: parameter elevation relationships on independent slopes model
*R*^2^: coefficient of determination
