# Supplementary material for: Multiscale divergence between Landsat- and lidar-based biomass mapping is related to regional variation in canopy cover and composition
Source: Carbon Balance Manag. 2018 Sep 14;13:15. doi: 10.1186/s13021-018-0104-6 (PMC6138055; doi:10.1186/s13021-018-0104-6)
Supplement: Supplementary file 1 — Additional file 1. Additional details regarding ecoregion descriptions, Landsat-based biomass modeling, and lidar-based biomass modeling. [file 13021_2018_104_MOESM1_ESM.docx]

**Multiscale divergence between Landsat- and lidar-based biomass mapping is related to regional variation in canopy cover and composition.**

David M. Bell, Matthew J. Gregory, Van Kane, Jonathon Kane, Robert E. Kennedy, Heather M. Roberts, Zhiqiang Yang

**Additional file 1: Additional details regarding ecoregion descriptions, Landsat-based biomass modeling, and lidar-based biomass modeling details**

*Ecoregion descriptions*

Across the study regions, individual ecoregions vary in terms of mean climatic and vegetation characteristics (Figure S1). Climatic and vegetation characteristics vary both between study regions and within study regions. This variation represents broad eco-climatic gradients across which remote sensing is utilized for mapping AGB.

*Landsat-based biomass modeling*

The development of LTS-based AGB maps included three primary stages: forest structure data extraction, environmental data extraction, CCA modeling and imputation. Plot-level tree data, in this case live tree basal area (m^2^ ha^-1^) by species and diameter class, were extracted from existing forest inventory plot systems, including Forest Inventory and Analysis Program [1] and Continuous Vegetation Survey [2]. These plots all utilized nested sampling designs that represent forest structure and composition over an area similar to a 3-by-3 Landsat pixel footprint (0.81 ha). For example, FIA plots in our study area combine four 14 m^2^ microplots (all trees ≥2.54 cm diameter at 1.37 m height), four 168 m^2^ subplots (all trees ≥12.7 cm diameter at 1.37 m height), four 1012 m^2^ macroplots (all trees ≥76.2 cm or 61.0 cm diameter at 1.37 m height west and east of the Cascade Mountains, respectively). For each plot, estimates of AGB were calculated based on the component ratio method [3] to be used for imputation after modeling.


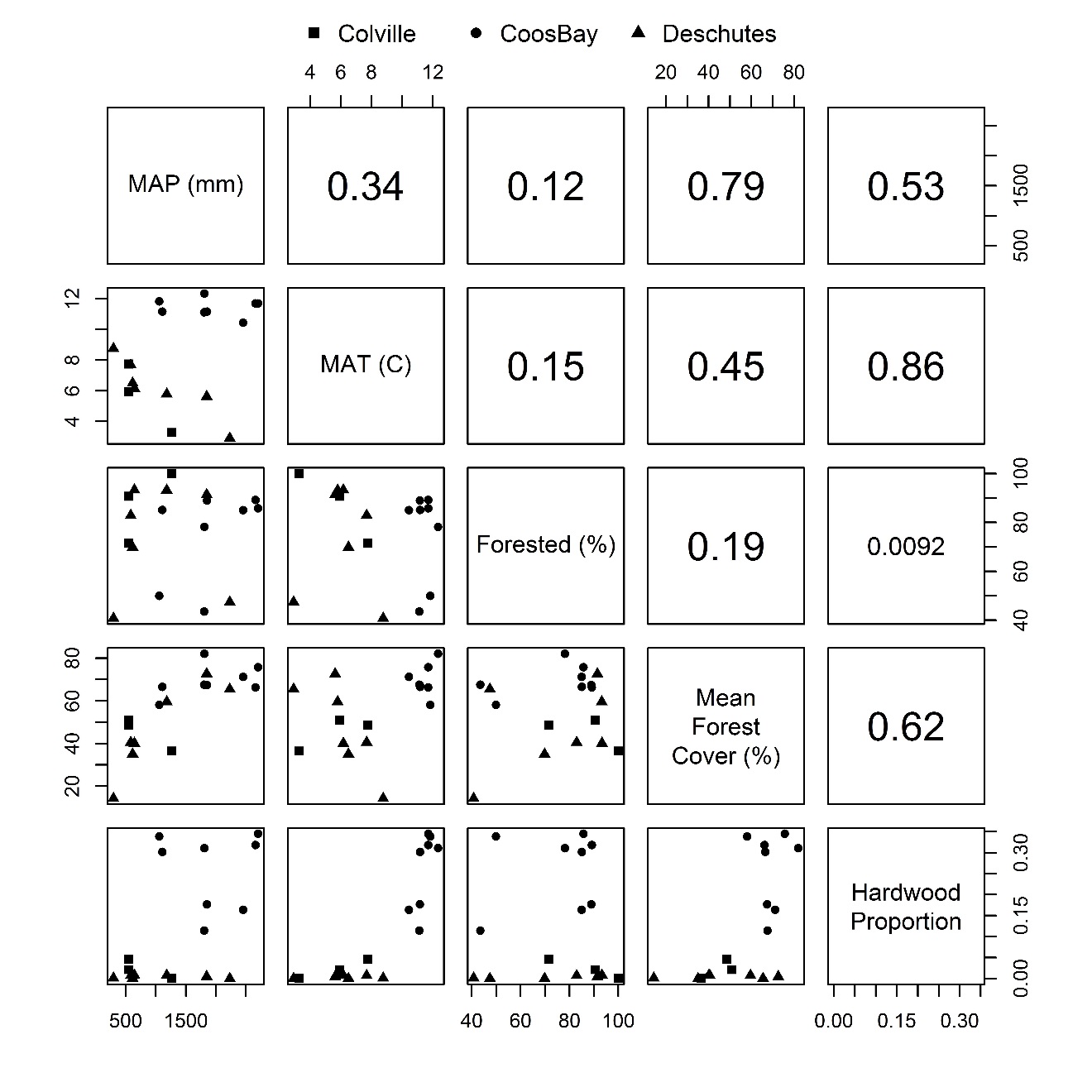


**Figure S1.** Variation in mean climatic conditions (mean annual temperature, or MAT, and mean annual precipitation, or MAP) and vegetation (percent forested, percent hardwood within forest, and canopy cover from FIA observations) conditions across ecoregions examined in this study. The lower triangle shows mean values for each ecoregion. The upper triangle shows the Pearson correlation coefficients.

Environmental data, both remote sensing and abiotic variables, were used as predictor variables in the GNN framework and were extracted from wall-to-wall geospatial layers (i.e., geospatial predictors), allowing for landscape mapping. We developed temporally smoothed, annual, Landsat TM and ETM+ image data processed through the LandTrendr algorithm [4]. LandTrendr is a trajectory-based change detection method that identifies fitted line segments of consistent trajectory for each pixel that describe sequences of vegetation disturbance and growth based on a spectral index [5]. Here, we used tasseled-cap greenness, brightness, and wetness [6] and NBR, extracting spectral indices for the year of plot measurement.

To account for variation in the relationship between LTS data and forest attributes across diverse landscapes, we incorporated measures of climate, topography, and location (resampled to the 30-m Landsat grid) into GNN modeling. For climate, log mean annual precipitation, mean annual temperature, mean maximum August temperature, mean minimum December temperature, and the ratio of growing season (May – September) temperature to log precipitation were resampled to 30 m from the PRISM using 800-m 30-year normal (1971-2000) mean monthly precipitation and temperature [7]. Topographic variables, including cosine transformed aspect, elevation, potential relative radiation, percent slope, and topographic position index (i.e., difference between elevation and mean elevation within 450 m), were resampled to 30-m pixels from the 10-m National Elevation Dataset [8]. Location information included longitude, latitude, and distance to the Pacific Ocean (i.e., coastal proximity). Thus, the geospatial data represent both the annual variation in LTS and the geographic variation in environmental gradients that might mediate the relationships between LTS and forest attributes [9].

We extracted all geospatial predictors for each plot footprint, here defined as the 3-by-3 pixel footprint centered on the forest inventory plot center. The geospatial data for the 3-by-3 pixel footprint (*n* = 9) were averaged to produce a plot-level representation of the LTS and environmental data. To minimize the chances of a temporal mismatch between forest inventory data and remote sensing, geospatial data were extracted for the year of plot measurement. At this 90-m level, geolocation errors associated with portable GPS units have minimal effects on map accuracy [10].

To define the gradient space used for identifying neighbors for imputation, CCA modeling was performed based on species matrices (live tree basal area by species and diameter class) and an environment matrices (geospatial data) [9]. The species matrix was constructed using the plot data mentioned above, but with plots in certain conditions removed: plots that straddled vegetation condition boundaries (e.g., harvest boundaries) or where the date of plot measurement and LTS acquisition straddled a disturbance were excluded from further analysis.

The CCA models, and the associated canonical variates, were used to orient both plots and pixels within the gradient space using *k* nearest neighbor (*k*NN) imputation with *k*=1. For each pixel (where AGB is unknown), the nearest plot (where AGB is known) in gradient space was identified and the associated AGB was imputed to the pixel, resulting in wall-to-wall AGB predictions for all pixels and all years from 1984-2012 (e.g., Fig. 1). Only the first eight axes were used to define the gradient space. We assumed zero AGB in non-forest portions of the landscapes, as indicated by land cover data from the USGS Gap Analysis Program (<http://gapanalysis.usgs.gov/gaplandcover/>) [11].

*Lidar-based biomass modeling*

Lidar-based biomass maps were based on remotely sensed data collected by Watershed Sciences, Inc. (WSI, Corvallis, OR; later merged into Quantum Spatial) (Table S1) and associated field data (Table S2), including individual tree diameter measurements and species identifications. We processed the lidar data supplied by the contractors using the USDA Forest Service's FUSION software package (beta version derived from version 3.2, http://forsys.cfr.washington.edu/fusion.html) [12]. All vegetation analyses were done with lidar return elevations normalized to height above ground to reflect height of vegetation canopy above ground. We used the contracter-supplied ground models which were produced using the TerraScan and TerraModel software packages (Terrasolid, Helsinki, Finland). We produced a set of vertical forest structure and canopy cover metrics from the lidar return using the FUSION software (Table S2). For each plot, we extracted the lidar returns above that plot and produced metrics using the FUSION cloudmetrics tool. We mapped the same metrics across the extent of the acquisition using a grid cell area the same as the plot area using the FUSION gridmetrics tool.

**Table S1**. Attributes of LiDAR acquisitions used in this study as reported by the contractors

| **Acquisition** | **Colville** | **Dechutes** | **Coos Bay** |
| --- | --- | --- | --- |
| **Dates Acquired** | 16-24 Apr 2008 & 05 Sept 2008 | Fall 2009,  Spring to Fall 2010 | June 2008 to April 2009 |
| **Contractor** | WSI | WSI | WSI |
| **Instrument** | Leica ALS50 Phase II | Leica ALS50 Phase II and ALS60 | Not reported^1^ |
| **Mean pulse density m^2^** | 7.2 | 8.6 | 8.1 |
| **Scan angle** | ±14° | ±14° | Not reported^1^ |
| **Survey altitude** | 900 m | 900 & 1300 m | Not reported^1^ |
| **Accuracy (RMSE, cm)** | 2.4 cm | 4 cm | 5 cm |
| **Max. returns per pulse** | 4 | 4 | 4 |

^1^Based on other acquisitions by the vendor in this time frame, likely to be similar to the Colville & Deschutes acquisitions

**Table S2**. Summary of information on field plot data collection for the study areas

| **Site** | **Field data collection** | **GPS coordinate collection** | **Plot number** | **Plot sizes** |
| --- | --- | --- | --- | --- |
| Colville | Data collected to support analysis of LiDAR data | Forest Service field crew | 157 | 0.08 ha |
| Deschutes | Data collected to support FIA plot remeasurements | Forest Service field crew | 303 | 4 0.17 ha subplots per plot |
| Coos Bay | Data collected to support analysis of LiDAR data | UW field crew | 893 | 0.05 ha |

For each study region, we modelled biomass using multiple linear regression models. We selected the list of possible explanatory LiDAR predictors from the metrics produced by the FUSION cloudmetrics program (Table S3). For some metrics, a 2-m threshold was used to exclude understory returns often unassociated with trees, such as ground, rocks, coarse woody detritus, and shrub and non-woody vegetation, which do not contribute to the field plot observations of live tree aboveground biomass. We examined every combination of one, two, or three explanatory variables using the regsubsets function in the leaps R package (version 3.0) [13] to identify the three models with the highest coefficient of determination (*R*^2^). Using this reduced set of candidate models we repeated this process three times: (1) testing interaction terms of explanatory variables, (2) after taking the square root, cube root, or logarithm of the response variable (whichever produced the most normal distribution based on the powerTransform function in the car package version 2.1-4)[14], and (3) considering both a transformed response and products of the explanatory variables.

**Table S3**. Metrics computed by the FUSION LiDAR processing software and tested for inclusion in models in this study [12].

| FUSION computes these following statistics using return height above ground elevation. Returns <2 m height were not used in the calculation of the height metrics. |
| --- |
| AAD (Average Absolute Deviation) |
| Coefficient of variation |
| Interquartile distance |
| Kurtosis |
| L-moment kurtosis |
| L-moment skewness |
| L-moments (L1, L2, L3, L4) |
| MADMedian (Median of the absolute deviations from the overall median) |
| MADMode (Median of the absolute deviations from the overall mode) |
| Maximum |
| Mean |
| Mean, cubic |
| Mean, quadratic |
| Median (output as 50th percentile) |
| Minimum |
| Mode |
| Percentile values (1st, 5th, 10th , 20th, 25th, 30th, 40th, 50th, 60th, 70th, 75th, 80th, 90th, 95th, 99th percentiles) |
| Skewness |
| Standard deviation |
| Variance |
| Cover |
| FUSION computes these cover metrics using the ratio of returns above a height break (e.g., 2 m or mean height) to the total number of returns |
| Cover >2 m (first returns) |
| Cover above mean height (first returns) |
| Cover above mode height (first returns) |
| Cover >2 m (all returns) |
| Cover above mean height (all returns) |
| Cover above mode height (all returns) |
| % all returns >2m/first returns |

These steps resulted in 36 models, from which we eliminated from consideration any which had any insignificant (*p*≥0.05) coefficients. We then performed the Breusch-Pagan test [15] to test for heteroscedasticity, the Ramsey Regression Specification Error Test [16] to test whether any of the predictors had a non-linear relationship with biomass density, and the Rainbow test [17] for model lack of fit (all tests done using the lmtest R package version 0.9-35) [18] on the remaining models. Where possible, we considered only models for a given study area that passed all three tests. For the Deschutes study region, this was not possible, and we considered only models that failed just one test. Finally, from these remaining models, we chose the one with the lowest Akaike Information Criterion [19] score to be our final model.

Multiple linear regression models for aboveground live tree biomass for each study region are reported in table S4. For all three study regions, the best model utilized a cubic transformation of the regression function as well as interaction and ratio terms, highlighting non-linearity in the relationships between lidar-derived metrics of forest structure and aboveground live tree biomass. NRMSE was low (≤18%) and *R*^2^ was high (≥0.69) for all three study regions, indicating good model performance. The resulting linear models were then predicted mean aboveground live biomass over the each LiDAR acquisition for rasters with grid cell size equal to the size of the plots within that acquisition (e.g., Figure. S2).

**Table S4**. Descriptions of lidar-based biomass maps

| **Study Area** | **Best Model** | **R ^2^** | **NRSME** |
| --- | --- | --- | --- |
| Colville | (1.891 + 0.033×*H*_90_ - 0.002×*V*:*CV* + 0.001×*P*_>2m_×*H*_95_)^3^ + 4.9 | 0.74 | 10 |
| Coos Bay | (2.322 + 0.038×*L*_1_ + 0.013×*P*_>2m_)^3^ + 35.47 | 0.69 | 18 |
| Deschutes | (0.748 + 0.036×*H*_70_ + 0.038×*Q*_>2m_)^3^ + 3.4 | 0.80 | 13 |

*H*_70_, *H*_90_, and *H*_95_ = 70^th^, 90^th^, and 95^th^ percentile height (m) of returns more than 2 m above the ground, respectively; *V:CV* = ratio of height variance to coefficient of variation in heights; *Q*_>2m_ and *P*_>2m_ = proportion first returns and all returns, respectively, greater than 2 m above the ground to the number of first returns; *L*_1_ = first L-moment of the height.


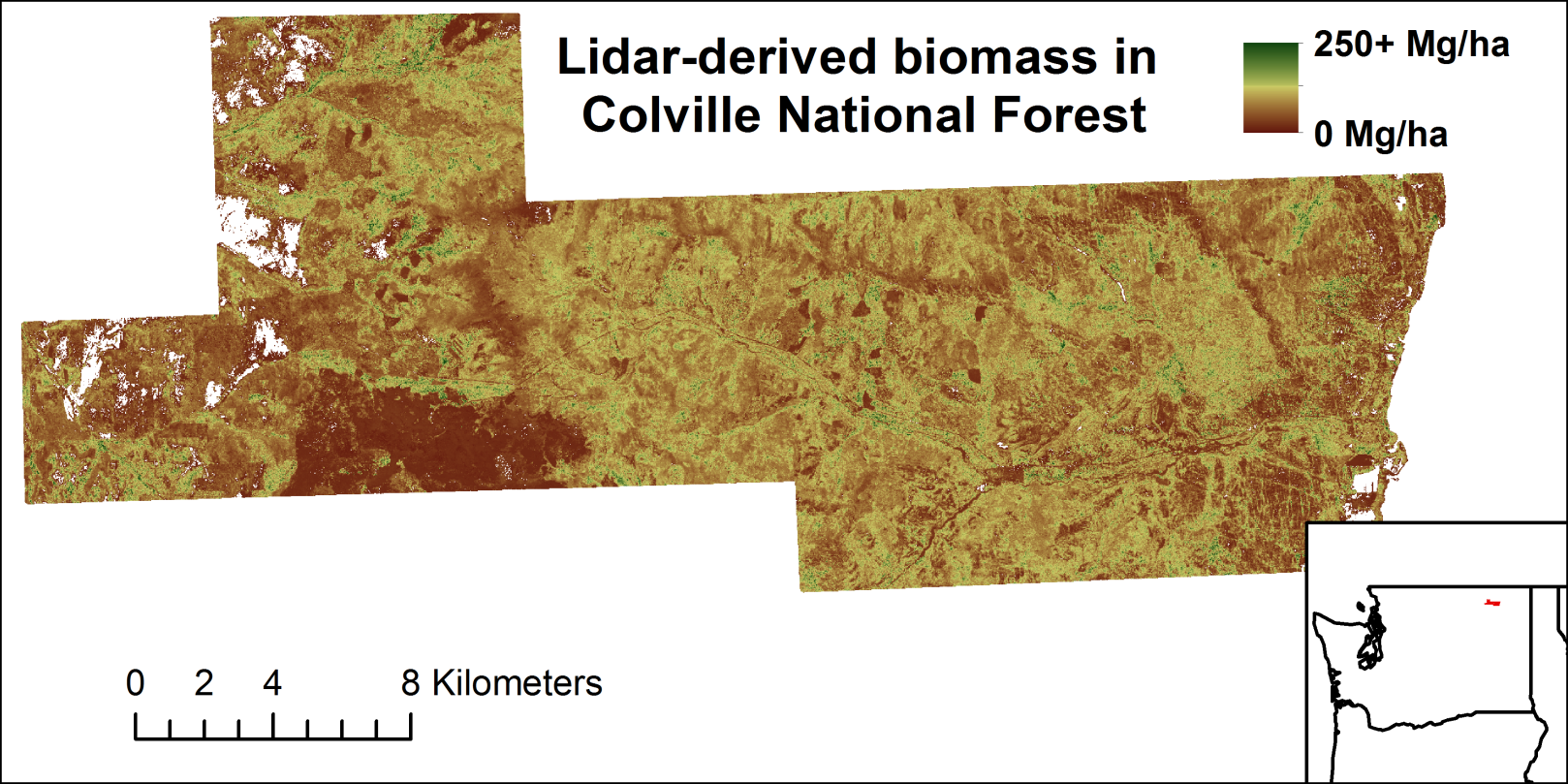


**Figure S2**. Example of the LiDAR modeled aboveground live tree biomass for the Colville study area. Blank area within the study are locations where no vegetation was >2 m in height.

**Literature Cited**

1. Bechtold WA, Patterson PL (2005) The Enhanced Forest Inventory and Analysis Program — National Sampling Design and Estimation Procedures. USDA Gen Tech Rep. doi: 10.2737/SRS-GTR-80

2. Max TA, Schreuder HT, Hazard JW, Oswald DD, Teply J, Alegria J (1996) The Pacific Northwest Region vegetation and inventory monitoring system. Res. Paper. PNW-RP-493. Portland, OR. USDA, Forest Service, Pacific Northwest Research Station. 22 p.

3. Heath LS, Hansen MH, Smith JE, Smith B, Miles PD (2009) Investigation into calculating tree biomass and carbon in the FIADB using a biomass expansion factor approach. McWilliams, W.; Moisen, G.; Czaplewski, R., comps. 2008 For. Invent. Anal. Symp. 2008 Oct. 21-23; Park City, UT. Proc. RMRS-P- 56CD. Fort Collins, CO U.S. Dep. Agric. For. Serv. Rocky Mt. Res. Sta

4. Kennedy RE, Ohmann J, Gregory M, et al (2018) An empirical, integrated forest biomass monitoring system. Environ Res Lett 13:041001

5. Kennedy RE, Yang Z, Cohen WB (2010) Detecting trends in forest disturbance and recovery using yearly Landsat time series: 1. LandTrendr — Temporal segmentation algorithms. Remote Sens Environ 114:2897–2910

6. Crist EP, Cicone RC (1984) A physically-based transformation of Thematic Mapper data --- The TM Tasseled Cap. IEEE Trans Geosci Remote Sens GE-22:256–263

7. Daly C, Halbleib M, Smith JI, Gibson WP, Doggett MK, Taylor GH, Curtis J, Pasteris PP (2008) Physiographically sensitive mapping of climatological temperature and precipitation across the conterminous United States. Int J Climatol 28:2031–2064

8. Gesch D, Oimoen M, Greenlee S, Nelson C, Steuck M, Taylor D (2002) The national elevation dataset. Photogramm Eng Remote Sensing 68:5–11

9. Ohmann JL, Gregory MJ (2002) Predictive mapping of forest composition and structure with direct gradient analysis and nearest- neighbor imputation in coastal Oregon, U.S.A. Can J For Res 32:725–741

10. Zald HSJ, Ohmann JL, Roberts HM, Gregory MJ, Henderson EB, McGaughey RJ, Braaten J (2014) Influence of lidar, Landsat imagery, disturbance history, plot location accuracy, and plot size on accuracy of imputation maps of forest composition and structure. Remote Sens Environ 143:26–38

11. Grossmann EB, Kagan JL, May HK, Gregory MJ, Tobalske C (2008) Final report on land cover mapping methods, map zones 2 and 7, Pacific Northwest ReGAP. Institute for Natural Resources, Oregon State University, Corvallis, OR.

12. McGaughey RJ (2016) FUSION / LDV : Software for LIDAR Data Analysis and Visualization, FUSION Version 3.60+.

13. Lumley T. using Fortran code by Miller A. (2017) Leaps: regression subset selection. R package version 3.0. http:// CRAN.R-project.org/package=leaps. http:// CRAN.R-project.org/package=leaps

14. Fox J, Weisberg S (2016) car: Companion to applied regression. R package version 2.1-2. https://cran.r-project.org/package=car.

15. Breusch TS, Pagan AR (1979) A Simple Test for Heteroscedasticity and Random Coefficient Variation. Econometrica 47:1287

16. Ramsey JB (1969) Tests for Specification Errors in Classical Linear Least-Squares Regression Analysis. J R Stat Soc Ser B 31:350–371

17. Utts JM (1982) The rainbow test for lack of fit in regression. Commun Stat - Theory Methods 11:2801–2815

18. Hothorn T, Zeileis A, Farebrother, R. W., Cummins, C., Millo, G., Mitchell D (2017) lmtest: Testing Linear Regression Models. R Package Version 0.9-34. https://CRAN.R-project.org/package=lmtest.

19. Akaike H (1974) A New Look at the Statistical Model Identification. IEEE Trans Automat Contr 19:716–723
